# A simplified courtship conditioning protocol to test learning and memory in Drosophila

**DOI:** 10.1016/j.xpro.2022.101572

**Published:** 2023-01-11

**Authors:** Beatriz Gil-Martí, Celia G. Barredo, Sara Pina-Flores, Adriana Poza-Rodriguez, Gaia Treves, Carmen Rodriguez-Navas, Lucía Camacho, Atenea Pérez-Serna, Iñaki Jimenez, Laura Brazales, Javier Fernandez, Francisco A. Martin

**Affiliations:** 1 Cajal Institute, Spanish National Research Council (CSIC), Av Dr Arce 37, 28002 Madrid, Spain; 2 Department of Biology, Autonomous University of Madrid, Madrid, Spain

**Keywords:** Behavior, Model Organisms, Neuroscience

## Abstract

In *Drosophila*, a male that has previously been sexually rejected reduces its courtship behavior when confronted again with an unreceptive female. This reduced courting time reflects a memory formation process. Here, we describe a simplified protocol to perform the courtship conditioning assay for assessing the reduced courting time, using regular lab equipment and handmade tools. Every step of the procedure, from raising flies and training to testing and quantification of this memory-related behavior, can be implemented in any practice laboratory.

## Highlights

• A simple and robust learning protocol that can be set up using ordinary lab supplies
• Requires minimal experience in *Drosophila* handling or behavioral assays
• Includes videos showing the procedure for training, testing, and quantification
• Possibility of using manual or automatized (supervised learning) quantification

## Abstract (editor highlights)

Publisher’s note: Undertaking any experimental protocol requires adherence to local institutional guidelines for laboratory safety and ethics.

## Graphical abstract

**Figure:**
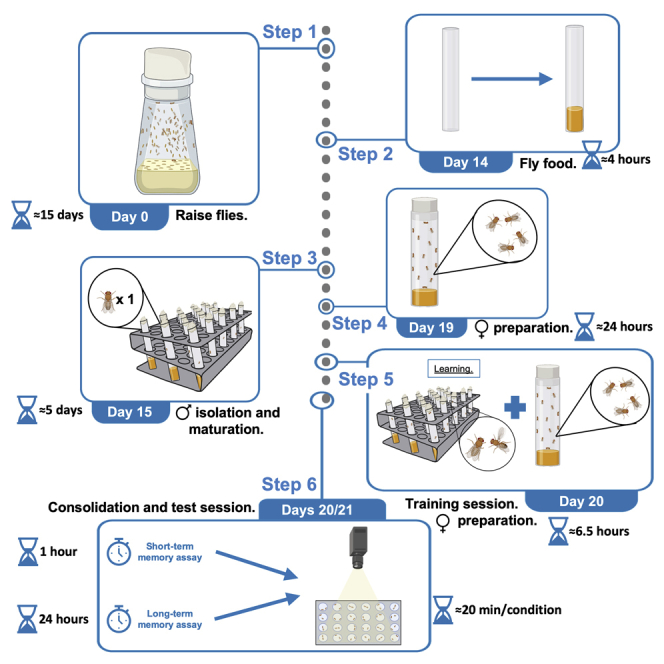

## Before you begin

### Prepare fly food, fly-containing vials and test arenas

**Timing: 2 days**

**Figure 1:**
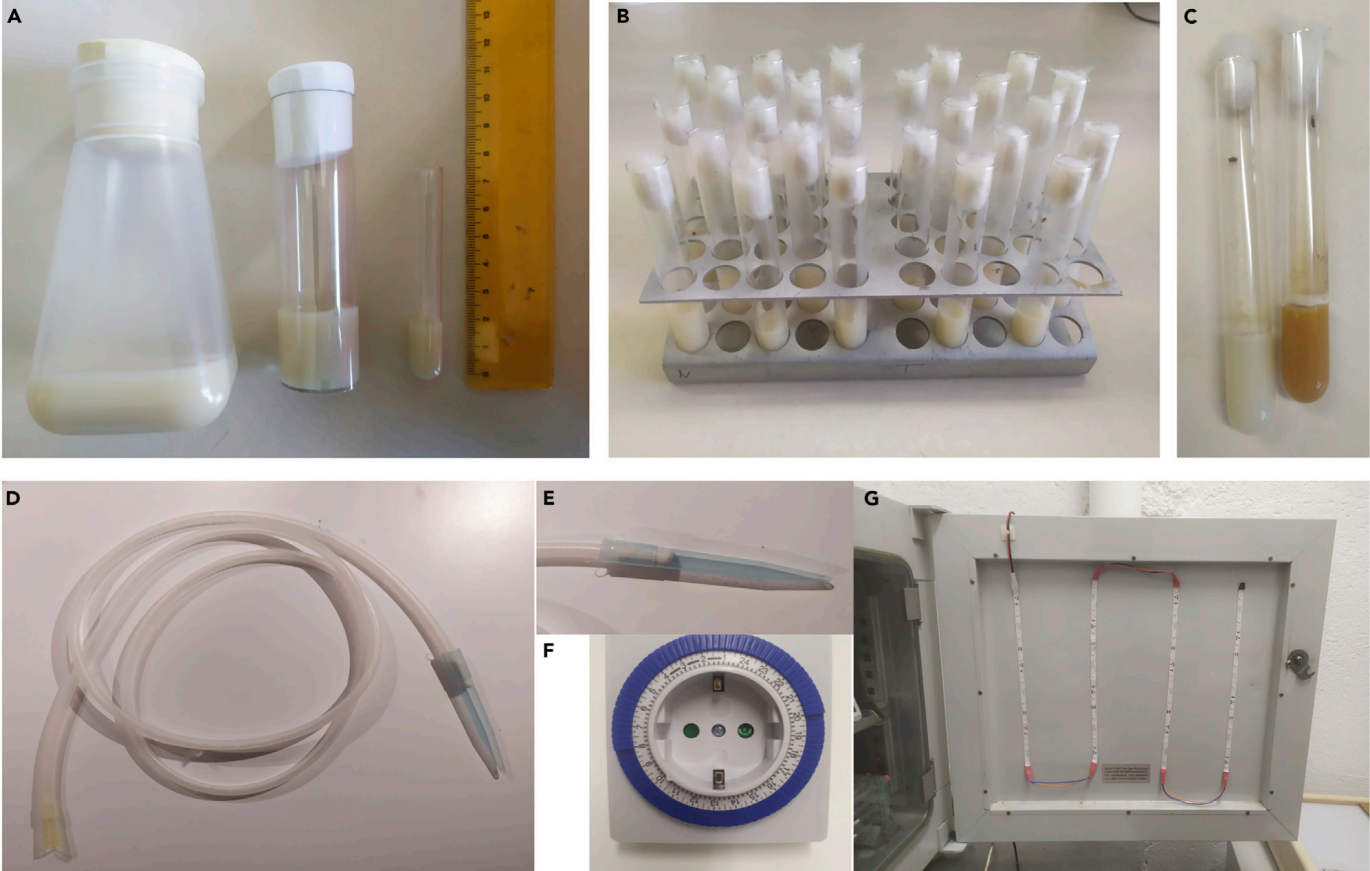
Before you begin (A) Examples of plugged bottle, vial and pyrex tube filled with fly food. (B) Disposition of pyrex tubes containing isolated flies in racks to avoid interaction. (C) Example of bacterial infection in a pyrex tube. (D) Homemade neumocaptor. (E) A detail showing the p1000 tip with the cotton. (F) An example of a commercial plug timer. (G) Customized incubator with LED strips on the door.
3. Close the bottles and tubes with ultra-dense plugs.

***Note:*** Maintain the fly food at 4°C up to 1 month or until they become dry.

1. Make 1 liter of fly food (see fly food recipe table below).
  a. Hydrate 26 g of dry yeast with 100 mL of water and autoclave (day 1).
  b. The next day (day 2), add this 100 mL of hydrated dry yeast extract, 54 g of Glucose, 36 g of wheat flour and 8.2 g of agar up to 1 L of water in this order while it is heating up to 100°C.
  c. Stir continuously. Cook for 20 min at 100°C.
  d. Let the solution cool down to 55°C and add 4.5 mL of 99% propionic acid.
2. Pour the food into bottles, vials and pyrex tubes. The height of the food should be 1 cm for bottles (ø × H: 57 × 103 mm) and pyrex tubes (ø × H: 12 × 75 mm) and 2 cm for vials (50 mL) (Figure 1A). Keep pyrex tubes in a closed box at 4°C (do not plug them with cotton). Cover bottles and vials with filter paper and let the food dry for 3 h at room temperature and then overnight (16–18 h) at 4°C.

### Prepare experimental mature adult males

**Timing: 15 days**

4. Raise the flies with the adequate experimental genotype for 15 days in bottles using an incubator at the adequate temperature (usually at 25°C, but 17° and 29° can be also used) with a 12:12 Light:Dark regime, although variations are admitted (16:8 L:D).

***Note:*** The genotype can be wild type (*Canton S*) flies, mutant animals or offspring from crosses (for instance, *Gal4/UAS* system).

***Note:*** For collecting 25–30 flies/day 2 bottles per genotype are enough, containing 22 females and 11 males. Alternatively, use 2 vials (with 11 females and 6 males) instead of one bottle.

**CRITICAL:** Avoid overcrowding by shifting flies every 2–3 days.

5. Collect experimental *Drosophila* males between 0–4 h post-eclosion and keep individually in *pyrex* tubes at 25°C for 5 days (or 9 days at 17°C) for a complete brain maturation.
6. Place tubes separately to avoid visual contact between flies (Figure 1B).

***Note:*** A final number of 15–20 males/condition should be enough to observe effects on memory. Because some of the flies will be discarded during training, 25–30 flies/condition would be a good number to start with.

***Note:*** Use CO_2_ or ether anesthesia to collect flies at this step.

***Note:*** Plug Pyrex tubes using cotton wool.

**CRITICAL:** Neither mites nor bacterial/fungi infection are admitted (Figure 1C).

### Prepare mature mated (gravid) adult females

**Timing: 1 h (1 day before training or testing)**

7. 24 h before training or testing, collect Wild Type females (*CS* strain) in a bottle or vial with WT males to promote mating.
8. Before training session starts, collect gravid females (virtually all females) and separate them from males in a tube (20 females per vial).

***Note:*** The proportion is 1:1, and no more than 60 animals per bottle (30 if using vials).

***Note:*** Use CO_2_ or ether anesthesia to collect (and count) males and females of 1-to-6 days old. Typically, collect gravid females 2 h before training (recovery from CO_2_ anesthesia requires one hour in the vial).

***Note:*** At least 24 h (ideally 48 h) are needed to ensure that all females have mated. Basically, all sexually mature females (older than 8 h post-eclosion) that are in contact with males will mate almost immediately, especially with a 1:1 ratio. In 24 h 100% of female flies mated successfully.

**CRITICAL:** Do not use *white* mutant females: they reject males less vigorously than CS or other wild type females.

### Institutional permissions

This protocol doesn’t need any institutional permission further than *Drosophila* handling.

## Key resources table

**Table:** 

REAGENT or RESOURCE	SOURCE	IDENTIFIER
**Chemicals, peptides, and recombinant proteins**		

Dry yeast extract (anchor yeast)	Duo Harinero	07402
Glucose	PanReac AppliChem	141341-0416
Wheat flour for bread	Duo Harinero	01256
Agar	Condalab	1804.05
99% propionic acid	Merck	8.00605.1000

**Experimental models: Organisms/strains**		

Drosophila melanogaster/*Canton S* - males, 5 days-old	Bloomington Stock Center	# 64349
Drosophila melanogaster/*dunce*^*1*^ - males, 5 d-o	Bloomington Stock Center	# 6020
Drosophila melanogaster/*rutabaga*^*2080*^ - Males, 5 d-o	Bloomington Stock Center	# 9405

**Software and algorithms**		

FlyTracker	Barwell et al. (2021)	https://github.com/kristinbranson/FlyTracker
JAABA	Kabra et al. (2013)	http://jaaba.sourceforge.net

**Other**		

Incubator∗	Any (Memmert, Heraeus)	N/A
LED strips∗	Any (Solbright)	N/A
Plug timer∗	Any (Garza)	N/A
Bottles∗∗	DD Biolab	789022B
Vials∗∗	Corning	FLY50-01
PYREX™ Disposable Round-Bottom Rimless Glass Tubes∗∗	Fisher Scientific	PYREX™ 99445-12
24-well cell culture plates∗∗	Falcon	353047
Laboratory racks∗∗	Any	N/A
Cotton wool∗∗	Any	N/A
Drosophila Anti-Mite Vial Plugs (Droso-Plugs®)∗∗	Genesee Scientific	59–200, 59–201, 59–194
Brush∗∗	Any	N/A
PLA Plastic for 3D impression. Transparent color (If OPTION B)∗∗	Any	N/A
Video Camera∗∗∗	Sony (min 30 FPS)	HDR-CX405-Videocamera
Tripod∗∗∗	DURAGADGET	Compatible Tripod for HDR-CX405
3D printer∗∗∗	Any (Ultimaker)	(Ultimaker S2 Extender Plus)
LED Light Lamps (If OPTION A)∗∗∗	Any (IKKEA)	(Nävlinge)
Transilluminator (If OPTION B)∗∗∗	Handmade	N/A
Computer (If OPTION B)∗∗∗	Any (requirements as in software-describing papers)	N/A

∗ Incubator with LED incorporated (handmade).

∗∗ Expendable Material.

∗∗∗ Video Recording Tools.

## Materials and equipment

**Table:** Fly food recipe

Reagent	Final concentration (p/V)	Amount
Hydrated yeast	2.6%	100 mL (26 g)
Glucose	5.4%	54 g
Wheat flour	3.6%	36 g
Agar	0.82%	8.2 g
dH_2_O	n/a	up to 1 L
**Total**	**n/a**	**1000 mL**

***Note:*** Maintain the fly food at 4°C up to 1 month or until they become dry.

**CRITICAL:** Add the reagents in this order while it is heating up to 100°C. Stir continuously. Cook for 20 min at 100°C. Let the solution cool down to 55°C and add 4.5 mL of 99% propionic acid.

To collect flies, *Drosophila* labs generally use CO_2_ and flypads, but 4°C works as an alternative if the protocol is implemented in a non-*Drosophila* lab. Placing the animals into a fridge for a while (until they do not move) and putting them on a pad within a box containing ice (or a cold pack) allows fly manipulation (under a stereomicroscope, if necessary). Most steps of this procedure do not require the use CO_2_ or ice, because both of them impede memory formation. Instead, employ a neumocaptor for handling flies. This is a handmade gadget: a p1000 tip to catch flies with cotton to avoid their inhalation and a p100 tip to aspire them fitted in both extremes of a flexible rubber tube (Figures 1D and 1E).

A quiet place with high humidity and good illumination (but no more than 2000 luxes) is essential for the success of this assay. In our experience, flies don’t learn or consolidate memory when they are disturbed. We commonly store flies in regular incubators (better climate chambers, but if not any can be adapted) where LED strips are stuck on the door and connected to an analogic plug timer, thus adjusted to a 12:12 Light:Dark regime. It is very easy to install; no special requirements or companies are needed. Actually, we have customized incubators that use materials from different suppliers (Figures 1F and 1G for examples).

In our hands, humidity is also very important and should be maintained between 50%–80% during training and consolidation. To obtain a humidity of 70% during consolidation, put an open-air box filled with saturated salty water inside the incubator. Regarding training or testing, a humidifier for a quiet room will create such wet environment. Illumination of animals during training is done by using commercial LED lamps with no further needs.

Temperature can oscillate (between 19°C and 27°C), but it is important that learning, consolidation and testing are performed at similar temperatures with only slight differences (2°C–4°C). Most experiments will be performed at 25°C. A detail that should not be oversighted is to do the experiment at the same circadian time. We start training and testing 2 h after light on (10:00 am, the moment of the day when learning is maximum in our hands).

The *Drosophila* Anti-Mite Vial Plugs can be substituted by other plugs employed in any Drosophila lab, such as rayon or cotton, in the case of bottles or vials. However, to cover the 24-well plate white foamed (cut or handmade) use plugs that fit in the 24-well plate (see Figure 3A).

## Step-by-step method details

In the supplemental information, there is a video showing crucial steps of training and testing session (Methods video S1, related to steps 3, 5–7 and 10–13).

The courtship conditioning or conditioned courtship suppression assay was described for the first time in *Drosophila melanogaster* in 1979 (Siegel and Hall, 1979). Since then, the knowledge about this process has been greatly increased and it is summarized in detail by Raun et al. (2021). The more generalized training for this protocol used to be three spaced 2-h trainings with 30 min resting intervals (Barajas-Azpeleta et al., 2018), although a single training of 5–7 h work similarly (Koemans et al., 2017). The latter protocol requires specific equipment and it is routinely employed by two different labs (Chubak et al., 2019; Lei et al., 2022), This learning generates a robust long-term memory, lasting at least 5–9 days (Brenman-Suttner et al., 2020; Inami et al., 2021). As other long-term memories, it is anesthesia sensitive (CO2 or ice cold). This memory is mainly generated by olfactory cues, but also involved visual inputs (Raun et al., 2021). Avoid the use of vision- or smell-deficient mutant animals (for instance, white). Also, the strength of the female genotype is important for the rejection of the male: in our hands, CS mated females render a 15% of mating males whereas white mated females have a 30%–35% of successful mating.

### Training session

**Timing: 6,5 h**

This section describes the preparation and the training (i.e., learning) of male flies in order to induce learning and/or to generate memory (either short- or long-term). The aim is to select male flies that have been rejected by females from courting (see Methods video S1).

***Note:*** Prepare Mated *Canton S* females for Test in a tube with males (1:1 proportion), approximately 24 h before Test session start.

**CRITICAL:** For training and testing sessions, avoid the use of CO_2_ on experimental males. Use a neumocaptor instead (see Figures 1D and 1E).

1. Get pyrex tubes containing 5-day old naïve (unmated) males.
2. PREPARATION:
  a. Train 2/3 of the males (around 20 animals).
  b. Place the pyrex tubes where they got mature in racks, trying not to cover other tube so the researcher can observe them without manipulating (Figure 2A).

**Figure 2:**
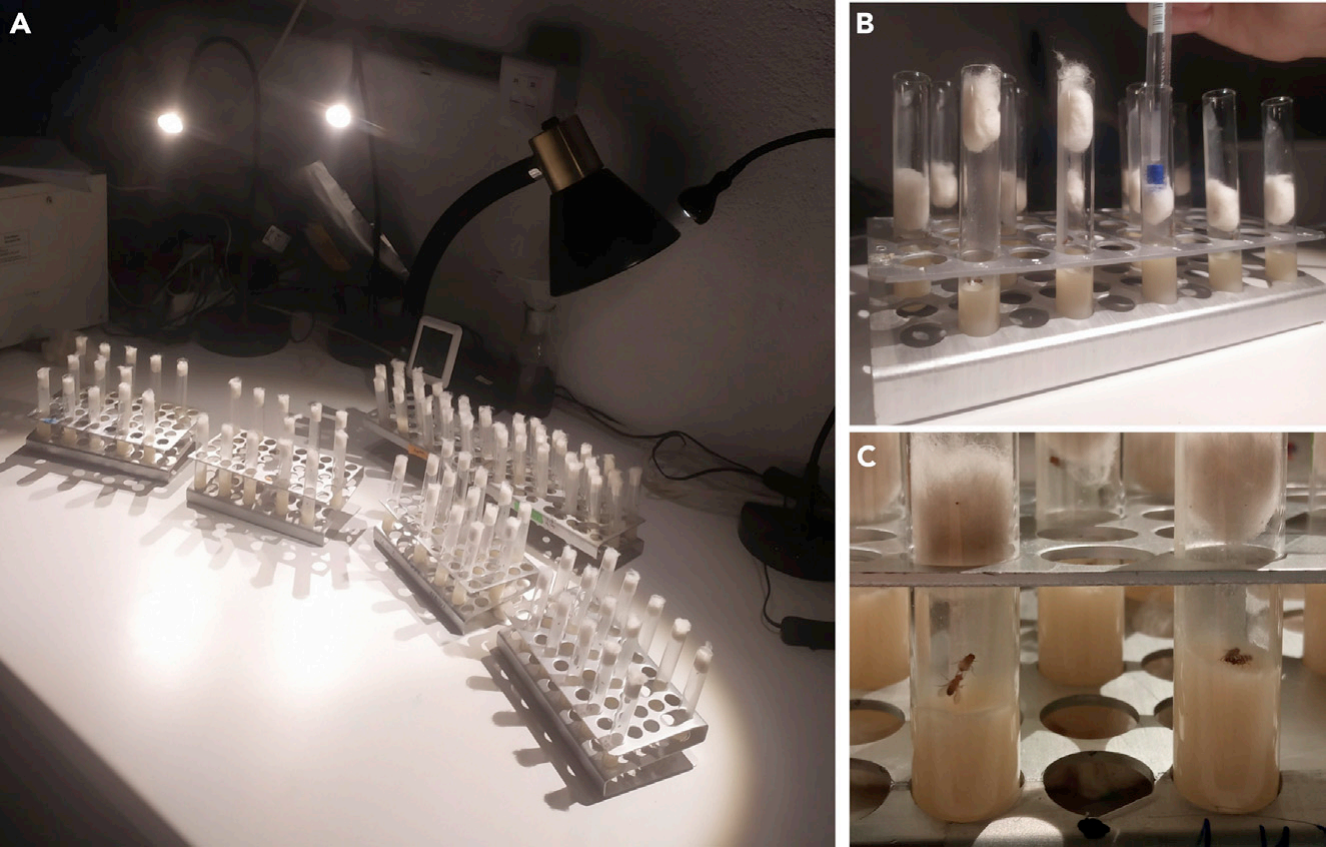
Training session (A) Example of the training session. (B) Cotton plug pushed down in order to promote fly interaction. (C) Two pyrex tubes containing two *Drosophila* couples. In the left one the male is doing the courtship, whereas in the right tube the male is actually mating.
  c. Place the remaining 1/3 of flies in in racks nearby, to experience the same environmental conditions. These will serve as naive, non-trained control flies.
3. TRAINING:
  a. Introduce a mated female by aspiration in the tube of each male that is going to be trained (using the neumocaptor).
  b. Once all tubes have a female, push the cotton plug near to the bottom down leaving a space of approximately 1.5 cm, in order to promote interaction. It can be done with the help of a forceps or with a pen. Try to push all the surface of the cotton to avoid flies from getting stuck between the plug and the tube wall (Figure 2B).
  c. Observe flies every 15 min without disturbing and mating couples discarded from the experiment (Figures 2C and 2D). *Drosophila* mating lasts 20 min, at least.
  d. Remove wool cotton after 5 h of training cotton and retire kindly the female through aspiration.
  e. Put the cotton back, leaving the male alone in the pyrex tube.

**CRITICAL:** From this step on it is very important not to disturb experimental males. Examples of disturbing are switching lights on/off, loud noises, vibrations of the table where the racks are and rapid temperature changes.

**CRITICAL:** Temperature and humidity should oscillate between 19°C and 27°C and 50%–80%, respectively.

***Note:*** In our hands, a successful training using CS means around a 15% of mating males or above. Other female genotypes may render different ratios, given that this percentage depends on the “strength” of females.

4. Different phases of memory formation can be studied with this protocol.
  a. LEARNING: Perform the test immediately or within a 15 min interval after the training session.
  b. SHORT-TERM MEMORY: Perform the test 1 h after training.
  c. LONG-TERM MEMORY: Perform the test 6–8 h after training (this is considered long-term memory). We typically do the assay 24 h after training. This requires a period of consolidation. Leave the males in their own tube in a temperature and humidity-controlled incubator for memory consolidation until test session starts.

**CRITICAL:** For long-term memory assays, it is very important not to disturb the experimental males during the critical period of consolidation. Examples of disturbing are opening/closing the incubator, altering the circadian rhythmicity, loud noises or vibrations by hitting the incubator.

Methods video S1. A video showing crucial steps of the step-by-step method section, related to steps 3, 5–7 and 10–13)

### Test session

**Timing: 30 min/condition**

This section describes how to retrieve the generated memory using a very simple test assay.

**CRITICAL:** Use a room without any source of illumination but the one used in your experiments. The dark room diminishes distracting visual cues, something that may affect the test performance. Also avoid speaking or disturbing animals in any way.

***Note:*** Test arenas are 24-well plates and they can be reused. Please do not use soap, just running water.

OPTION A (for traditional analysis).

This option is recommended for researchers that are not going to use assiduously the protocol or do not have the possibility to use an Automatized Tracking Program. Quantify manually the obtained video.

**Figure 3:**
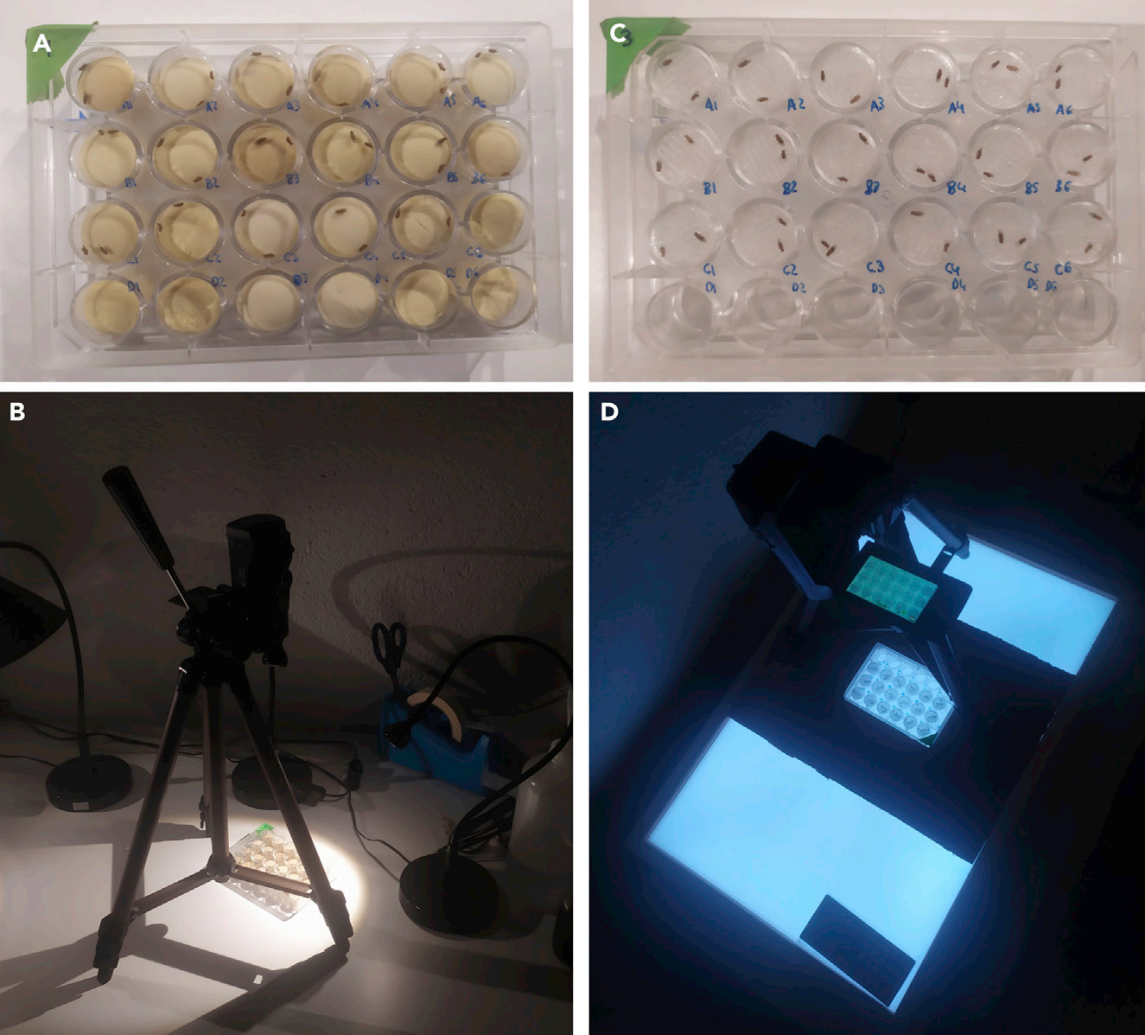
Testing session (A) The plugged 24-well plate with animals included, ready to be recorded. (B) Example of the set up. (C) The 24-well plate with the transparent Tapino lids over the flies. (D) Example of the set up.
8. Record courtship behavior. Obtain a 10 min video at least (Figure 3B).
9. Discard flies once they are recorded or keep if needed.

***Note:*** For white high-dense plugs, we cut the *Drosophila* Anti-Mite Vial plugs used for vials. Alternatively, commercial white foam can be cut to proper size.

5. Introduce a male in each well (24 flies in a 24 wells plate) by aspiration with white high-dense plugs. Test the naïve males too.
6. Introduce a mated female in each well with the male.
7. Push plugs down in order to create a 2D space (Figure 3A).

OPTION B (for automatized analysis).

This is the best option because it allows the use of an Automatized Tracking Program, which makes quantification much easier and less human-working time consuming. The weak point is that the tracking software may be not easy to implement.

10. Aspirate a female and a male and together introduce them in a well of the 24-well plate.
11. Cover the well with the *Tapino* (3D-2D) lid and introduce the aspirator from one side. Test naïve males too.
12. Press the *Tapino* lid until the bottom to create a 2-dimensional space which is highly important for the posterior tracking and analysis of the flies (Figure 3C).
13. Cover the plate with its own lid and flip it to check if all the flies are correctly placed and *Tapino* lids well situated.
14. Place the plate in the transilluminator, always in the same position.
15. Record courtship behavior. Obtain a 10 min video at least (Figure 3D).

**Figure 4:**
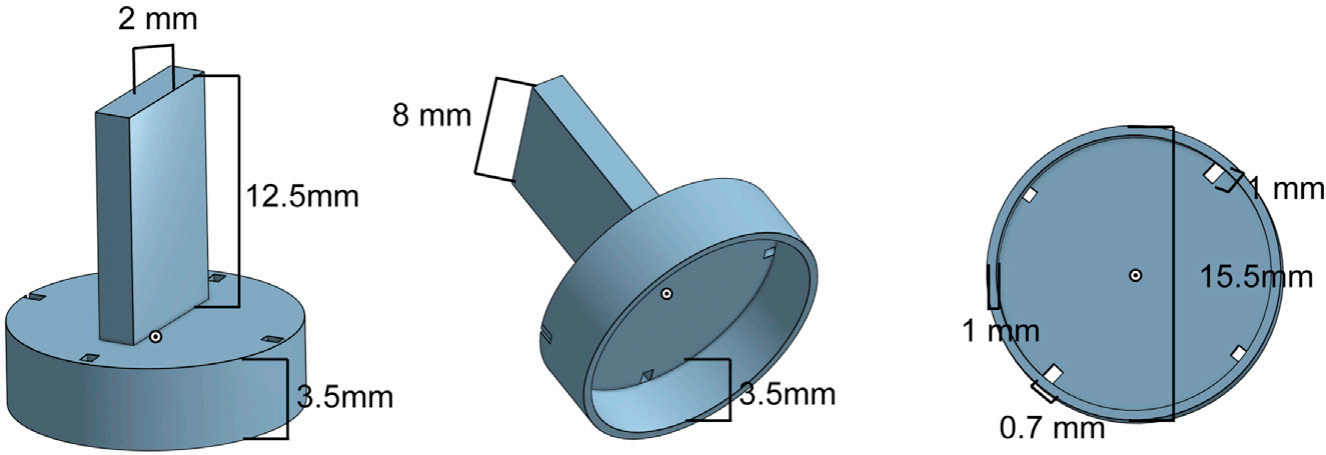
The *Tapino* lid scheme, including dimensions This 3D printed lid leaves a 3,5 mm space where flies can interact and behave comfortably, but in a 2-dimensional space that is a fundamental requisite for most automatic tracking programs.

***Note:*** To place the plate always in the same position, cut a piece of cardboard with the shape of the plate in the middle and place it in the transilluminator in such a way that it cannot move. Also mark the position of the tripod legs on the cardboard: the camera needs to be placed in the exact same position every time, otherwise illumination is modified and the posterior automatized analysis will not work properly (Figure 3D).

***Note:*** The *Tapino* lid is an in-house design and requires a 3D printer (Figure 4 and Data S1).

## Expected outcomes

**Figure 5:**
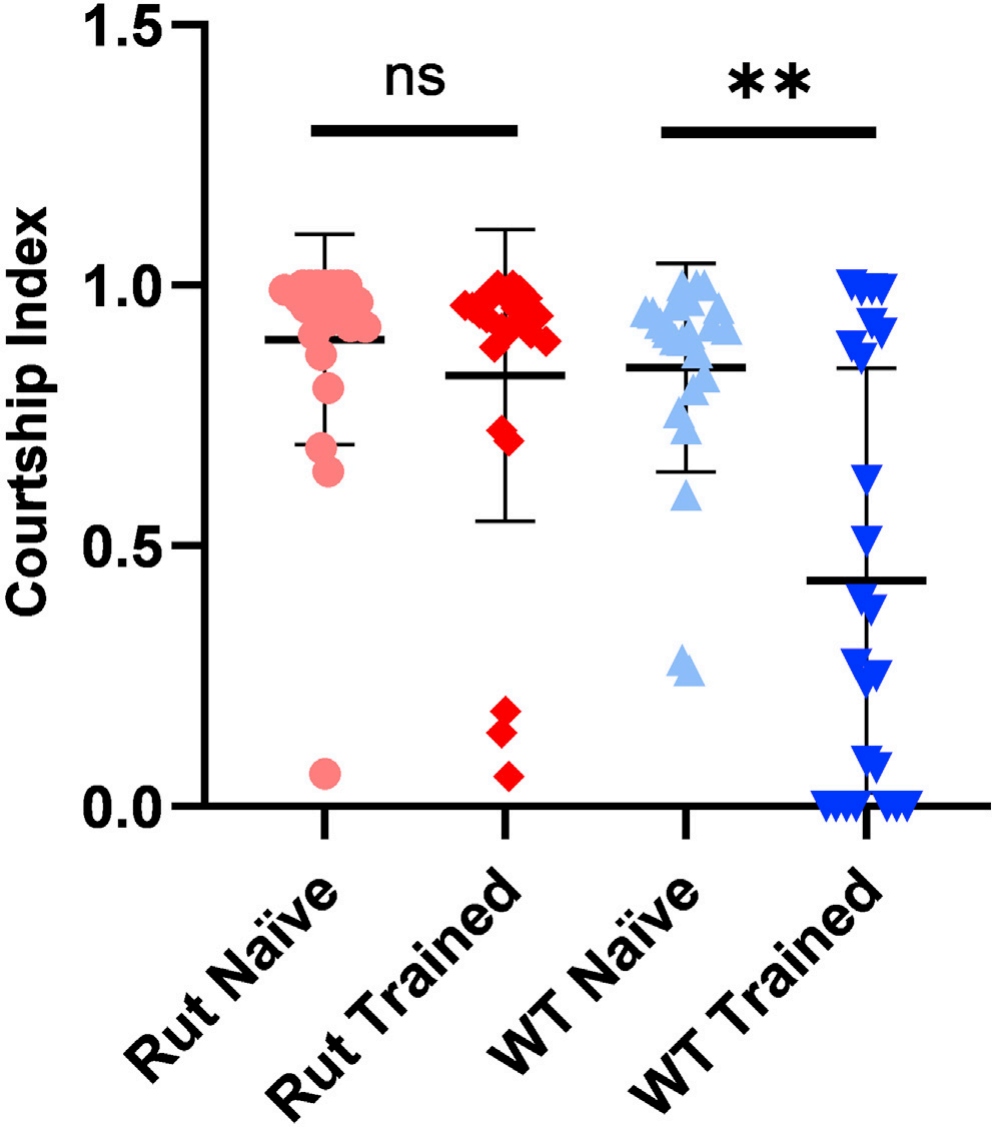
Representation of a long-term memory experiment Real data from two trials of rutabaga mutant and wild type flies (n=24, mean±SD) (Mann-Whitney U-test; ∗∗p value < 0.01).

During training, a good signal is that males are actually courting with some successful mating (15% approximately). Figure 5 represents real data of two trials together showing the typical distribution for a positive experiment, using *Canton S* and *rutabaga* (*rut*) mutant flies. *rut* encodes a Ca^2+^/calmodulin-activated adenylyl cyclase that is responsible for synthesis of cAMP, which is required to create both short- and long-term memory but not for learning. Virtually all animals lacking cAMP (and consequently, activated CREB) are unable to create memory (Kaldun and Sprecher, 2019). The difference between the CI of trained and naive CS flies are significant, whereas in the case of *rut* animals they are not (Mann-Whitney U-test). In our hands, usual numbers are around 0.8–1 for naive flies and around 0.5–0.6 for trained flies, but this vary greatly between experiments and labs.

## Quantification and statistical analysis

This section describes the two methods (manual and automatized) we have routinely employed in our lab to quantify the behavior. Also, we include the most widely used two statistical analyses for this assay.

### Quantification

#### Option A

This option is the simplest one and it does not require any further material. Once the 10 min video is obtained, quantify the time that the male spends doing courtship. There are several videos and reviews explaining the different phases of courtship (for instance, see Sokolowski, 2001; https://www.youtube.com/watch?v=zXXqQ2zJVMA for a video). In our lab, we follow the following criteria: the interest of the male in the female usually starts with a short wing flutter near to the female (up to two bodies away). It normally ends when the male tries to mount the female several times and she rejects him roughly. We don’t consider courtship the time that the male spends staring at the female from a certain distance (see Methods video S3 for an example of quantification). These courtship criteria can be modified by the researcher if always the same criteria are followed throughout all the experiments.

A graduate student trained for 10 min can easily learn how to quantify the courtship behavior of a male. Annotate the moment(s) in which the courtship begins and finishes, then sum up the duration of all attempts.

#### Option B

**Figure 6:**
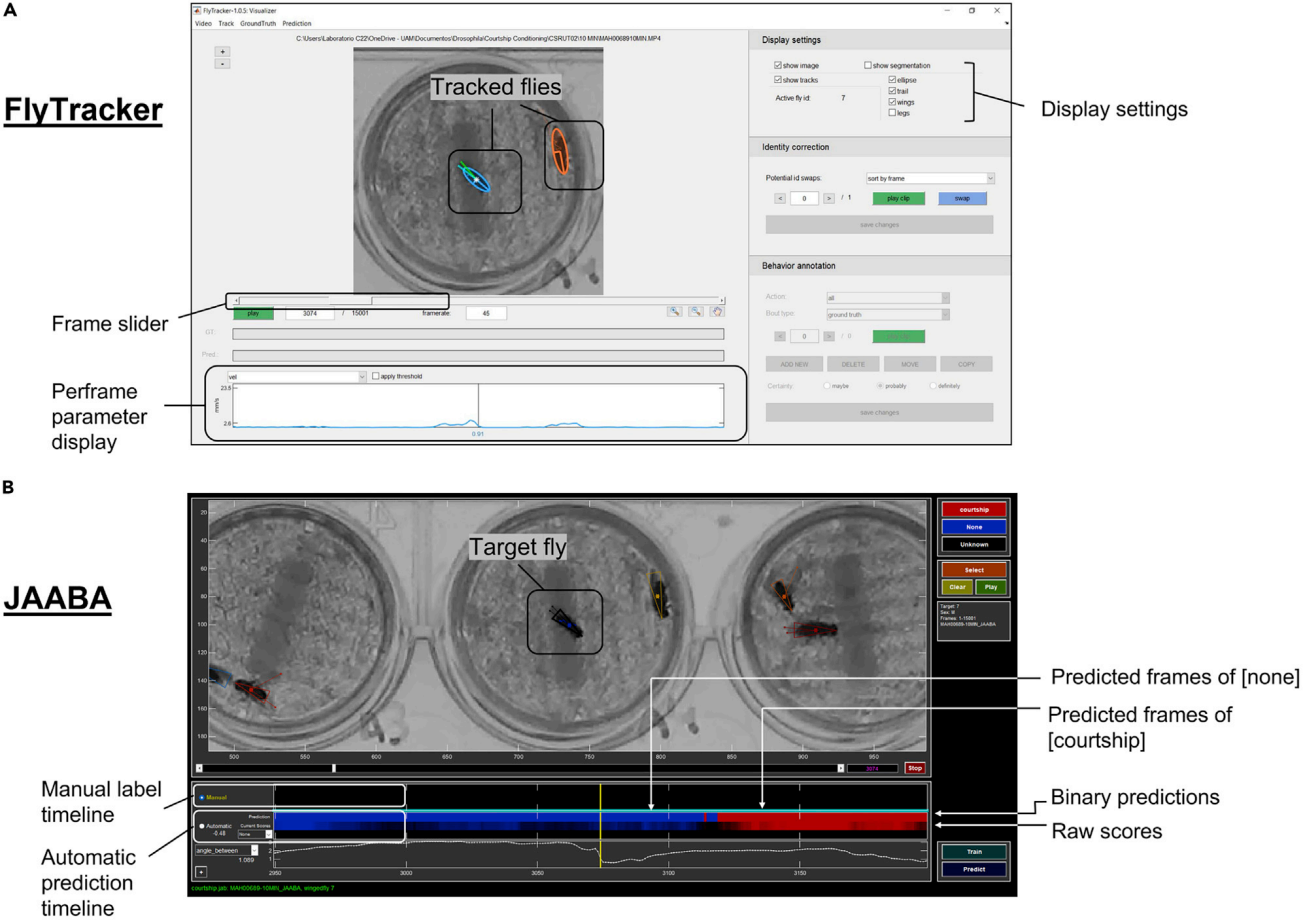
Automatized quantification (A) Annotated interface of the FlyTracker software. (B) Annotated interface of JAABA software.

This is an automatic process. It takes less time for the researcher but requires to record videos under with the same conditions, using a transilluminator and the *Tapino* lids (see Data S1). These *Tapino* lids are transparent and force the flies to move in a two-dimensional space (Figure 2). Different tracking software are available such as Ctrax, idTracker or Cadabra (Dankert et al., 2009; Pérez-Escudero et al., 2014; Stern et al., 2015). In our hands, FlyTracker MATLAB combined with JAABA works fine, but any of the others yields a good result (Barwell et al., 2021; Kabra et al., 2013). Figure 6 shows a screenshot of both software interfaces. In Methods videos S2 and S3 there is an example of the Fly Tracker tracking and the subsequent automatized courtship quantification by JAABA, respectively. It should be noticed that JAABA requires supervised learning that can be applied only to the experimental conditions of each lab.

Methods video S2. 1-min video showing the tracking of a male and a female during the test session (Fly Tracker), related to quantification section (option B)

Methods video S3. The same 1-min video showing the automatized quantification of JAABA after supervised training of the software, related to quantification section (option B)

### Statistical analysis

The courtship index (CI) is defined as the percentage of time that the male courts during the first 10 min of the testing period divided by 100, for each individual male fly. The simplest way to analyze data is to represent the mean CI of naive and trained flies for each genotype. If there is a significant difference (by means of a Mann-Whitney U-test) then flies have successfully learned.

However, most authors prefer to use learning index (LI- also known as memory index) for representation, defined by the formula: LI = (CI naive – CI trained) / CI naive) = 1- (CI trained / CI naive), using the mean CIs. The statistical analysis of this LI is more complex and has been described elsewhere (Kamyshev et al., 1999; Koemans et al., 2017).

**CRITICAL:** To reinforce conclusions, repeat the experiment at least twice (better three times, n>40).

***Note:*** We also suggest to use *rutabaga* or *dunce* mutant flies (that cannot form memory) as a negative control.

## Limitations

As all behavioral paradigms, the main drawback is the relatively high number of times that the assay does not work. In order to learn, flies need to be in a calm state throughout the learning process.

For the study of aging and neurodegenerative diseases, a learning/memory assay like this might be of great interest. However, use cautiously this paradigm and test it previously with appropriate controls (i.e., young flies): it is still under debate whether or not aged flies have affected learning and short-term memory after courtship conditioning, which suggests that the genetic background might be important (Brenman-Suttner et al., 2020). Regarding long-term memory, no studies have been done so far.

Another limitation is that this is a population study, not per individual. Actually, in our experience not all trained flies diminished considerably their courtship behavior, just a percentage of them (see Figure 4A for data points distribution).

Whereas other paradigms have their equivalent on mice or rat assays (for instance, olfactory conditioning is similar to fear conditioning), still it is not clear what type of learning courtship conditioning is or its equivalence in a mammalian learning paradigm, if any (see (Raun et al., 2021).

## Troubleshooting

### Problem 1

Insufficient number of males to train (before you begin-prepare experimental males).

The number of emerged males of interest (to be trained) may be not enough to perform the experiment. Do not try to do the training with low number of animals because results will not be reliable.

### Potential solution

Use flies of 5-to-7 day-old altogether, there is no difference in learning among them. Another solution is to use additional bottles to increase the number of males, however this will require at least 10 days (*Drosophila* life cycle at 25°C).

### Problem 2

Few males mate (training session).

During the training, it is possible that not many males mate. In our hands, for CS a successful training means a 15% of mated females. If lower, it suggests that training did not work.

### Potential solution

Repeat the experiment and change some experimental conditions: for instance: use 7 h of training instead of 5 h; change the mated female genotype.

### Problem 3

Flies do not learn (test session/quantification).

To detect whether the training worked or not, always test positive control flies (i.e., CS naive and trained) simultaneously to the experimental samples. It is recommended to test negative control animals too (i.e., *dunce* or *rutabaga* mutant naive and trained animals). If Ci or LI in the positive control (naive vs trained sibling CS flies) does not render a significant difference, discard the experiment.

### Potential solution

Make sure the conditions are adequate. Quiet place, high humidity, 12:12 Light:Dark Cycle, always do the experiment at the same daytime. A small number of copulating pairs during training (below 5%) may indicate that males are under stress (i.e., do not courtship and consequently do not learn).

Also check the size of females used to train the males. If female animals are too small or weak they do not reject them with enough vigor. To solve it, obtain bigger females giving them more food access during larval stages (by putting less parental animals in the bottle or vial, or shifting them more frequently).

### Problem 4

Installing the software for automatic training (quantification).

Despite the software repository is usually very complete (pay especial attention to documentation) installation may be sometimes tricky.

### Potential solution

There are generally specific google groups (for JAABA, for instance) where you can make your question. Under our experience, authors from these software-describing papers are keen to solve any issue you have by e-mail.

### Problem 5

Non-significant differences (statistical analysis).

Sometimes CI differences between trained and naive populations are small and not render a statistically significant difference.

### Potential solution

The experiment may need to increase the number of flies and replicates. Indeed, if differences are still small but consistent and reproducible, then the effect of the experimental condition on memory can be trusted.

## Resource availability

### Lead contact

Further information and requests for resources and reagents should be directed to and will be fulfilled by the lead contacts, [Beatriz Gil-Martí] (bgmarti@cajal.csic.es) and [Francisco A Martin] (famartin@cajal.csic.es).

### Materials availability

The *Tapino* lid file for printing. Data S1.

## Data Availability

Data are available upon request. All codes used in this protocol are freely available.

## Supplemental information

Data S1. The *Tapino* lid file for printing, related to the step-by-step method section, step 11)
